# Age- and Sex-Related Differences in Force-Velocity Characteristics of Upper and Lower Limbs of Competitive Adolescent Swimmers

**DOI:** 10.2478/v10078-012-0026-4

**Published:** 2012-05-30

**Authors:** Pantelis Theo Nikolaidis

**Affiliations:** 1 Laboratory of Human Performance and Rehabilitation, Division of Physical and Cultural Education, Hellenic Army Academy, Athens, Greece.

**Keywords:** arms, legs, power output, speed, strength

## Abstract

While there is a direct relationship between maximal anaerobic power (Pmax) and swimming performance, the relationship between upper and lower limbs with regard to Pmax and force-velocity (F-v) characteristics is not clear. The aim of the present study was to examine the effect of age and sex on the ratios of mechanical characteristics between upper and lower extremities of adolescent swimmers. Seventeen girls (aged 14.7±1.8 yr) (mean±standard deviation) and 28 boys (14.6±1.4 yr), all members of competitive swimming clubs, performed a F-v test for both legs and arms. In legs, boys had higher values of Pmax (t_43_=2.4, p<0.05), Pmax expressed in relative to body mass values (rPmax, t_43_=3.4, p<0.01) and v_0_ (t_43_=4.3, p<0.001), while no differences were found for F_0_ (t_43_=1.0, p=0.31) and v_0_/F_0_ (t_43_=0.55, p=0.59). In arms, boys had higher values of Pmax (t_43_=3.2, p<0.01), rPmax (t_43_=3.9, p<0.001) and v_0_ (t_43_=3.4, p<0.01), while no differences were found for F_0_ (t_43_=1.9, p=0.06) and v_0_/F_0_ (t_43_=0.16, p=0.87). However, no sex difference was found with regard to the ratios of Pmax (t_43_=1.9, p=0.06), F_0_ (t_43_=1.2, p=0.23) and v_0_ (t_43_=1.3, p=0.20) between upper and lower extremities. There was direct relationship between age and Pmax of legs (r=0.64, p<0.01 in girls; r=0.43, p<0.05 in boys) and arms (r=0.56, p<0.05; r=0.57, p<0.01 respectively), while there was not any significant association between age and the ratios of mechanical characteristics of upper and lower limbs. These findings emphasize the need for separate evaluation of arms’ and legs’ force-velocity characteristics on a regular basis and the consideration of these measures in training design.

## Introduction

Performance in swimming depends on physiological and psychological characteristics of athletes. These characteristics in adolescent athletes are under the influence of growth and maturation and consequently they may be differed from those of their adult counterparts. Young swimmers do not differ only from their adult counterparts, but also from athletes of other sport disciplines; e.g. compared with tennis, figure skating and volleyball players, adolescent female swimmers have unique physiological characteristics of aerobic power, muscular endurance and flexibility (Leone et al., 2002). Compared with badminton players, archers and nonathletes, swimmers exchibit better muscle symmetry and increased symmetry of autonomic indices (Balashova et al., 2004). In addition, adolescent swimmers have more stable circulatory system than those, who are not engaged in sports (Luchitskaya and Rusanov, 2009), and they have lower heart rate at rest than basketball players and other athletes (Vanyushin and Sitdikov, 2001).

With regard to bioenergetics, all swimmers do not have a unique profile and the relative contribution of each metabolic pathway (ATP-CP, lactic anaerobic, aerobic) depends on swimming distance (Volkov et al., 2005). Hawley and Williams (1991) noted that time in swimming over 50 m was correlated with anaerobic power of arms. Subsequent investigators have shown that performance in 50 m was associated with anaerobic power of legs (Duché et al., 1993) and that performance in 25–100 m was correlated with both upper and lower limbs’ anaerobic power (Strzala and Tyka, 2009). While there are the abovementioned reports of significant correlation between performance in swimming and anaerobic power of upper and lower extremities, the relationship between arms and legs’ anaerobic power is less clear.

Given that it is a sport that engages both movements of upper and lower limbs, it is necessary to examine their corresponding physiological characteristics. Until now, most of the research about the relationship between upper and lower extremities’ characteristics has focused on parameters of cardiorespiratory power, like maximal oxygen uptake, aerobic power output, anaerobic threshold, work efficiency and oxygen kinetics. Vokac and co-workers (1975) during a study on male subjects noted that though the maximal workload in arm exercise was 50–60% of that in cycling, VO_2_ in arm work was at maximal effort only 22% lower than in leg exercise. Other investigators have shown that the anaerobic thresholds for arm cranking and leg cycling occurred at 47% and 64% of VO_2max_, respectively (Davis et al., 1976) and that metabolic efficiency as determined by work efficiency indices was lower during arm crank compared with cycle exercise at the same relative intensities (Kang et al., 1997). Finally, a study in oxygen uptake kinetics now demonstrates that the time constant of the fast component response is significantly longer and greater in arm exercise compared to leg exercise (Koppo et al., 2002).

On the other hand, less information with respect to anaerobic characteristics of upper and lower extremities is available. Detailed information about one’s anaerobic power can be obtained by valid and reliable laboratory methods, such as Wingate 30 s anaerobic test (Ayalon et al., 1974), Bosco 60 s test (Bosco et al., 1983) and Force-velocity (F-v) test (Vandewalle et al., 1985). With respect to the other tests, F-v test has an advantage, because it provides information not only about maximal power (Pmax), but also about the constituents of power, i.e. force and velocity. Our previous work, employing the F-v test and conducted on active male students, showed that the arms to legs’ ratio with regard to P_max_ was 0.65, in theoretical maximal force (F_0_) 0.63 and in velocity (v_0_) 1.09 (Nikolaidis, 2006). Respective values in kickboxers were 0.46, 0.57 and 0.83 (Nikolaidis et al., 2011), and in boxers 0.49, 0.61 and 0.81 (Giovani and Nikolaidis, 2012). Nevertheless, these ratios may be sport-dependent and under the effect of training, and therefore they should be examined separetely for each sport.

Separate arms and legs’ power output measures would be useful in evaluating training programs and in understanding the importance of power output for swimming performance. Whether upper to lower limbs ratios of F-v characteristics of adolescent swimmers depend on sex is not known. Moreover, it has not yet been determined whether these ratios are influenced by age. Therefore, in the present study, we have examined anaerobic power of both upper and lower limbs. Our goal was to test two related research hypotheses: a) there are sex differences with regard to mechanical characteristics between upper and lower limbs, and their ratios, and b) there is association between age and these ratios.

## Methods

### Participants and procedures

Seventeen girls, aged 14.7±1.8 yr, and 28 boys, 14.6±1.4 yr, all members of competitive swimming clubs, volunteered for this study (Table 1).

The local Institutional Review Board approved this study and oral consent was obtained by all participants’ parents, after a verbal and written explanation of the experimental protocol and its potential risks. Exclusion criteria included history of any chronic medical conditions and use of any medication. No current injury was reported. All participants visited once our laboratory, in which they were tested for anthropometric characteristics and body composition, and they performed the Force-velocity test for both legs and arms after a standardized 15-min warm-up.

### Equipment and protocols

Height and body mass were measured using a stadiometer (SECA, Leicester, UK) and an electronic scale (HD-351, Tanita, Illinois, USA), respectively. Percentage of body fat was calculated from the sum of 10 skinfolds using a skinfold calliper (Harpenden, West Sussex, UK), based on the formula proposed by Parizkova (1978). The employment of skinfolds as a method of body fat estimation was validated in a sample of 12–18 yr swimmers, where the correlation between skinfold thickness and dual-energy X-ray outcome was 0.98 (Tuuri and Loftin, 1999).

The F-v test was used to assess Pmax, v_0_ and F_0_, and it employed various applied braking forces that elicited different pedalling velocities in order to derive Pmax (Vandewalle et al., 1985). The warm-up activity, which was conducted before the test, included stretching exercises, steady-paced cycling, and short submaximal sprints. Minimal warming-up and learning experience was necessary in order to perform a true maximal sprint. The participants performed four supramaximal pedal sprints, each lasting 7 sec, against incremental braking force, on a cycle ergometer (Ergomedics 874, Monark, Sweden). During each sprint, participants were encouraged to reach their maximal velocity as soon as possible. Seat height was adjusted to each participant’s satisfaction, and toe clips with straps were used to prevent the feet from slipping off the pedals. The participants performed five supramaximal pedal sprints, each lasting 7 sec, against incremental braking force, on an arm-cranking and cycle ergometer (Ergomedics 874, Monark, Sweden).

**Figure 1:**
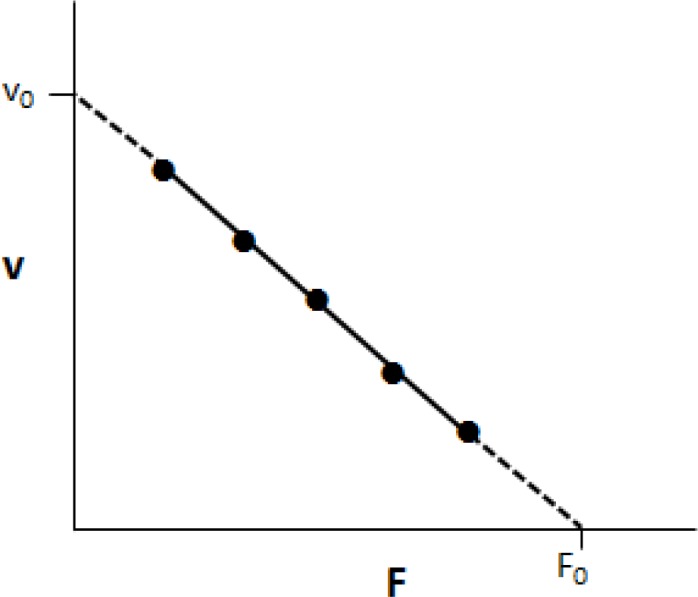
The inverse linear relationship between braking force (F) and velocity (v), and their corresponding theoretical maximal values (F_0_and v_0_)

The test began with a braking force of 30 N for legs and 20 N for arms. In every subsequent sprint, 10 N was added. During each sprint, participants were encouraged to reach their maximal velocity as soon as possible. This value of peak velocity was recorded and used to calculate F-v relationship (Figure 1).

The recovery period between each exercise bout was 5 minutes. Sprints were performed for legs and arms alternately. The F-v test was suggested to be reliable measure of short-term power output of children, adolescents and adults tested twice within a week (test-retest coefficient of variation 3% (Doré et al., 2003)). With regard to its validity, this test was highly correlated with the Wingate anaerobic test (Vandewalle et al., 1987).

### Data and statistical analysis

For each participant, an individual linear regression (least squares method) was determined between peak pedalling frequency and breaking force for each of the five sprints (five data points for each F-v relationship). The F_0_ and v_0_ corresponded to the intercepts with the force and velocity axes in the F-v graph. At both of these locations, power is equal to zero. Because both velocity and force are nonzero between these endpoints, power varied with a bell-shaped profile depending on the magnitude of the product (Enoka, 1994). Pmax was determined at an optimal force and optimal velocity of 0.5 F_0_ and 0.5 v_0_ and was calculated as Pmax = 0.25 · F_0_ · v_0_. The comparison for each measured parameter between upper and lower limbs was calculated by the equation $x=\frac{y}{z}$, where x was the result of comparison, y the upper limbs’ mean value and z the corresponding mean value of lower limbs. The duration of every flywheel revolution was measured with the help of electronic sensor and power output of every revolution was computed by specialized software.

All data are presented as means ± standard deviations. The Pearson product moment coefficient of correlation (*r*) was used to examine the association between upper and lower limbs with regard to F-v characteristics, as well as the relationship between age and these characteristics. The dependent one-tailed Student *t*-test was used to determine whether upper and lower limbs mechanical characteristics’ means differed from each other, and the independent *t*-test to examine sex differences. Statistical analyses were performed using IBM SPSS v.20.0 statistical software (SPSS Inc., Chicago, IL, USA). Significance was set at alpha =0.05 for all the tests.

## Results

The force-velocity characteristics of upper and lower limbs of participants are presented in Table 2. In girls, arms and legs differed with regard to Pmax (*t*_16_=14.4, *p*<0.001), rPmax (*t*_16_=19.8, *p*<0.001), F_0_ (*t*_16_=15.3, *p*<0.001), v_0_ (*t*_16_=47.5, *p*<0.001) and v_0_/F_0_ (*t*_16_=14.2, *p*<0.001). In boys, upper and lower extremities differed with respect to Pmax (*t*_27_=17.8, *p*<0.001), rP_max_ (*t*_27_=31.5, *p*<0.001), F_0_ (*t*_27_=19.8, *p*<0.001), v_0_ (*t*_27_=48.8, *p*<0.001) and v_0_/F_0_ (*t*_27_=20.1, *p*<0.001). All participants had lower values in arms than in legs, except of v_0_/F_0_.

In both upper and lower limbs comparable sex differences were found. In legs, boys had higher values of Pmax (*t*_43_=2.4, *p*<0.05), rPmax (*t*_43_=3.4, *p*<0.01) and v_0_ (t_43_=4.3, *p*<0.001), while no differences were found for F_0_ (*t*_43_=1, *p*=0.31) and v_0_/F_0_ (*t*_43_=0.55, *p*=0.59).

In arms, boys had higher values of Pmax (*t*_43_=3.2, *p*<0.01), rPmax (*t*_43_=3.9, *p*<0.001) and v_0_ (t_43_=3.4, *p*<0.01), while no differences were found for F_0_ (*t*_43_=1.9, *p*=0.06) and v_0_/F_0_ (*t*_43_=0.16, *p*=0.87). In addition, no sex difference was found with regard to the ratios of Pmax (*t*_43_=1.9, *p*=0.06), F_0_ (*t*_43_=1.2, *p*=0.23) and v_0_ (*t*_43_=1.3, *p*=0.20) between upper and lower extremities.

**Figure 2:**
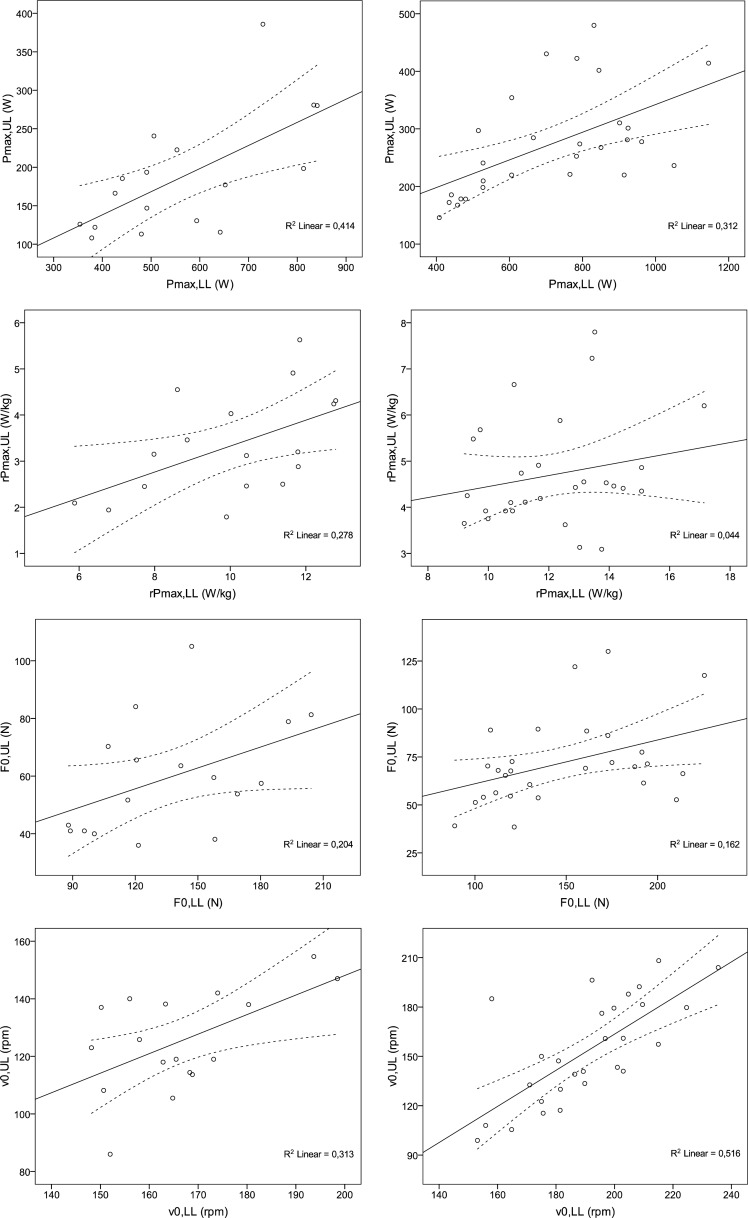
Relationship between upper and lower limbs’ mechanical characteristics in girls (left) and in boys (right). Dashed lines represent 95% confidence intervals of means

The mechanical characteristics of lower limbs were in association with the corresponding of upper limbs. In girls, these associations, with the exception of F_0_, were statistically significant; *r*=0.64 (*p*<0.01) in Pmax, *r*=0.53 (*p*<0.05) in rPmax, *r*=0.45 (*p*=0.069) in F_0_ and *r*=0.56 (*p*<0.05) in v_0_. In boys, these associations, with the exception of rPmax, were also statistically significant; *r*=0.56 (*p*<0.05) in Pmax, *r*=0.21 (*n.s.*) in rPmax, *r*=0.40 (*p*<0.05) in F_0_ and *r*=0.72 (*p*<0.001) in v_0_ (Figure 2).

As shown in Table 3, P_max_ was in direct relationship with age for both sexes and for both upper and lower limbs. Regarding the rest parameters of F-v relationship, there was no consistency in their association with age.

## Discussion

Although it is clearly recognized that anaerobic power is linked with performance in swimming, little is known about the F-v characteristics of those who practise this sport. This is the first study to examine the relationship between upper and lower limbs’ F-v relationship in swimmers. We demonstrated that Pmax, rPmax, F_0_, v_0_ and v_0_/F_0_ differed significantly between arms and legs. Pmax, rPmax, F_0_ and v_0_ were higher in legs, while v_0_/F_0_ was higher in arms, i.e. arms had a more “fast” profile and legs a more “strong” profile. These observations were noted in both sexes. With a few exceptions (F_0_ in girls and rPmax in boys), we observed direct relationships between upper and lower extremities’ mechanical characteristics, i.e. the higher the value of legs, the higher the value of arms, and vice versa. In girls, Pmax of legs accounted for by 41% of the variance in Pmax in arms and in boys, the respective value was 31%.

With regard to sex-related differences, either in upper or in lower extremities, boys had higher values of Pmax, rPmax and v_0_ than girls, while no differences were found for F_0_ and v_0_/F_0_. Previous studies had identified the higher power in boys. For instance, arm muscle power, assessed by Wingate anaerobic test, was higher in male swimmers than females (Ogonowska et al., 2009), while leg muscle power, assessed by various vertical jumps, was also higher in elite male swimmers than their female counterparts (Buśko and Gajewski, 2011). Age was in direct relationship with Pmax, but the association with the other measures of F-v parameters was not statistically significant. Our results were scrutinized together with relevant data of other researchers, who used similar methods. The positive relationship between age and Pmax came to terms with previous findings (Duché et al., 1993; Prioux et al., 2001; Vandewalle et al., 1989). Elite French male swimmers had upper limbs’ Pmax 286 W, rPmax 6.3 W·kg^−1^, v_0_ 206 rpm and F_0_ 55 N in age 12.5 yr, which were lower than the corresponding values of their 17.5 yr counterparts: 718 W, 10.1 W·kg^−1^, 254 rpm and 112 N (*n*=28) (Vandewalle et al., 1989). In another study on French male swimmers’ arms, aged 15.2 yr, v_0_ was 222 rpm, F_0_ 100 N, Pmax 565 W, rPmax 8.9 W·kg^−1^ and v_0_/F_0_ 2.45 rpm·N^−1^ (Prioux et al., 2001). In French male swimmers’ lower extremities, aged 11.3 yr, Pmax was 565 W and rPmax 8.9 W·kg^−1^ (Duché et al., 1993).

F_0_, 72 N and 148 N, of upper and lower limbs in boys respectively, is lower than the corresponding values in male students (140 N and 223 N (Nikolaidis, 2006)) and in active male adults (values only for lower extremities; 112 N (Vandewalle et al., 1985); 198 N (Chamari et al., 1995)). V_0_, 153 rpm and 191 rpm, of upper and lower extremities in boys accordingly, is also lower than previous findings for upper limbs (229 rpm in male students (Nikolaidis, 2006)) as well as for lower limbs (211 rpm in male students (Nikolaidis, 2006); 216 rpm in young endurance athletes ((Chamari et al., 1995); 228 rpm in recreationally active men (Vandewalle et al., 1985)).

The result of Pmax for upper limbs (272 W) is lower than the reference data (790 W (Nikolaidis, 2006), 884 W for 44 yr and 960 W for physical education students (Adach et al., 1999)). The corresponding values for lower limbs (708 W) is also lower than other reported data (1211 W (Nikolaidis, 2006), 1180 W in students (Jaskolska et al., 1999); 1114 W in 44 yr; 1029 W in physical education students (Adach et al., 1999); 1090 W in young endurance athletes (Chamari et al., 1995), 813 W in subjects with recreational activities (Vandewalle et al., 1985); 879 W in untrained students (Linossier et al., 1996)). The measured with the F-v test rPmax for upper limbs is 4.7 W·kg^−1^, while other studies reveal higher values (10.7 W·kg^−1^ (Nikolaidis, 2006); 10.7 W·kg^−1^ in 44 year-olds and 12.3 W·kg^−1^ in physical education students (Adach et al., 1999); 10.7 W·kg^−1^ in swimmers (Mercier et al., 1993)). The corresponding value for lower limbs (12.2 W·kg^−1^) is lower than previous reports; 16.4 W·kg^−1^ (Nikolaidis, 2006); 13.0 W·kg^−1^ in untrained students (Linossier et al., 1996); 13.2 W·kg^−1^ in physical education students, 13.7 W·kg^−1^ in 44 year-olds (Adach et al., 1999).

The ratio upper to lower limbs Pmax (0.40) is lower than the 0.65 (Nikolaidis, 2006), 0.78 in 44 year-olds and the 0.93 in physical education students (Adach et al., 1999). Two possible explanations for the discrepancy of our results in comparison with previous data (lower values in all the F-v characteristics) might be the age of participants and the sport. All the characteristics measured by F-v test (force, velocity and power) correspond to age-dependent sport-related fitness parameters (muscular strength, speed and anaerobic power).

Potential differences between arms and legs could be explained primarily due to muscle mass and muscle fibre type distribution. Muscle strength or force generating capacity is found closely related to muscle mass (Lanza et al., 2003; Metter et al., 2004) and muscle cross-sectional area (Maugha et al., 1984). It is proposed that upper limbs muscle mass is 22% (Abe et al., 2003) to 25% of lower limbs (Zatsiorsky, 2002). Our data additionally suggest that other factors, e.g. sport discipline in swimming, training, individualized technique and injuries, might also influence these differences. As shown in the Figure 2, there was a case of three female swimmers who had similar force in legs (120 N, 121 N and 122 N), but their corresponding force in arms differed (84 N, 66 N and 36 N) resulting in a wide range of ratio between upper and lower limbs (0.70, 0.54 and 0.30).

A drawback of our study was the inherent limitation of laboratory methods to reproduce the real movements of swimming. In addition, arms and legs’ power output was examined separately, which did not correspond to the complex movements of the sport that involve the coordination of upper and lower limbs. On the other hand, the laboratory methods provided valid and reliable measures of anaerobic power. Moreover, the distinction between arms and legs’ power came to terms with the training practice, in which many exercises, either in pool or in the gym, focus on specific body parts. A remarkable observation from the present study was the variability of the ratios of mechanical characteristics between arms and legs in swimmers. Based on these findings, it is recommended to monitor these characteristics regularly and to consider them in the training design.

This study attempted to quantify the proportionality of mechanical characteristics between swimmers’ limbs. The results confirmed previous observations that upper limbs had lower values of mechanical characteristics with respect to upper limbs. However, what is novel, is the quantification of these differences in the same participants, young competitive swimmers, which could have practical implications in aspects of their sport training. Further research in this topic should examine the association between swimming performance and the upper to lower limbs’ ratio in power output and F-v characteristics, in order to answer the question if there is any optimal ratio.

## Conclusions

This study was the first one to focus on differences of force-velocity characteristics between upper and lower limbs in competitive adolescent swimmers. In summary, we attempted to quantify the proportionality of mechanical characteristics (power, force and velocity) between swimmers’ upper and lower extremities. The results confirmed previous observations in general population that arms had lower values of power and force with respect to legs, and smaller differences concerning velocity. Our findings emphasize the need for separate evaluation of arms’ and legs’ force-velocity characteristics on a regular basis and the consideration of these measures in training design.

## Figures and Tables

**Table 1:** Anthropometric characteristics of participants

	Girls	Boys
BM (kg)	56.5±11.5	58.2±10.4
Height (m)	1.62±0.08	1.68±0.09^*^
BMI (kg·m^−2^)	21.5±3.2	20.6±2.4
WHR	0.73±0.03	0.79±0.03^‡^
BF (%)	22.8±5.7	14.5±4.1^‡^
FFM (kg)	43.1±6	49.5±8.4^†^

* p<0.05,

† p<0.01,

‡ p<0.001 (Student’s t test) denote differences between the two groups. BM is body mass, BMI body mass index, WHR waist-to-hip ratio, BF body fat and FFM fat free mass.

**Table 2:** Force-velocity characteristics of participants

		Girls	Boys
Lower limbs	Pmax (W)	565±162	709±210^*^
	rPmax (W·kg^−1^)	10.0±2.1	12.2±2.0^†^
	v_0_ (rpm)	166±14	191±21^‡^
	F_0_ (N)	136±37	148±40
	v_0_/F_0_ (rpm·N^−1^)	1.31±0.38	1.37±0.36
			
Upper limbs	Pmax (W)	188±76	272±90^†^
	rPmax (W·kg^−1^)	3.3±1.1	4.7±1.0^‡^
	v_0_ (rpm)	125±18	153±32^†^
	F_0_ (N)	59±20	72±22
	v_0_/F_0_ (rpm·N^−1^)	2.31±.78	2.36±.97
			
Upper to lower limbs ratio	Pmax	0.34±0.09	0.40±0.11
	F_0_	0.45±0.14	0.50±0.15
	V_0_	0.76±0.09	0.80±0.13

* p<0.05,

† p<0.01,

‡ p<0.001 (Student’s t test) denote differences between the two groups

**Table 3:** Correlation coefficient r between age and the force-velocity characteristics of participants

		Girls	Boys
Lower limbs	Pmax (W)	0.64^†^	0.43^*^
	rPmax (W·kg^−1^)	0.23	0.14
	v_0_ (rpm)	0.19	0.56^†^
	F_0_ (N)	0.60^*^	0.26
	v_0_/F_0_ (rpm·N^−1^)	−0.47 (*P*=0.06)	−0.09
			
Upper limbs	Pmax (W)	0.56^*^	0.57^†^
	rPmax (W·kg^−1^)	0.16	0.37 (*P*=0.05)
	v_0_ (rpm)	0.34	0.37 (*P*=0.06)
	F_0_ (N)	0.47 (*P*=0.06)	0.37 (*P*=0.05)
	v_0_/F_0_ (rpm·N^−1^)	−0.30	−0.08
			
Upper to lower limbs ratio	Pmax	0.06	0.23
	F_0_	−0.08	0.14
	V_0_	0.26	0.10

* p<0.05,

† p<0.01
